# Crop Contamination and Human Exposure to Per- and Polyfluoroalkyl Substances around a Fluorochemical Industrial Park in China

**DOI:** 10.3390/toxics12040269

**Published:** 2024-04-04

**Authors:** Kairan Xu, Jian Huang, Yufeng Zhang, Xilong Wu, Dan Cai, Guocheng Hu, Yu Li, Zhuobiao Ni, Qingqi Lin, Shizhong Wang, Rongliang Qiu

**Affiliations:** 1 Maoming and Heyuan Branch, Guangdong Laboratory for Lingnan Modern Agriculture, Guangdong Provincial Key Laboratory of Agricultural & Rural Pollution Abatement and Environmental Safety, College of Natural Resources and Environment, South China Agricultural University, Guangzhou 510642, China; xukairan@stu.scau.edu.cn (K.X.); huangjian_scau@foxmail.com (J.H.); zhangyufeng1214@foxmail.com (Y.Z.); caramel_ln@foxmail.com (X.W.); nizhuobiao@scau.edu.cn (Z.N.); qiurl@scau.edu.cn (R.Q.); 2 South China Institute of Environmental Sciences, Ministry of Ecology and Environment, Guangzhou 510655, China; huguocheng@scies.org (G.H.); liyu8@scies.org (Y.L.); 3 State Environmental Protection Key Laboratory of Environmental Pollution Health Risk Assessment, Guangzhou 510655, China; 4 State Environmental Protection Key Laboratory of Urban Ecological Simulation and Protection, Guangzhou 510655, China; 5 School of Environmental Science and Engineering, Sun Yat-sen University, Guangzhou 510006, China; wshizh2@mail.sysu.edu.cn; 6 Guangdong Provincial Key Laboratory of Environmental Pollution Control and Remediation Technology, Guangdong Provincial Engineering Research Center for Heavy Metal Contaminated Soil Remediation, Guangzhou 510006, China

**Keywords:** per- and polyfluoroalkyl substances (PFAS), novel fluorinated alternatives, crop contamination, health risk assessment

## Abstract

Due to their significant environmental impact, there has been a gradual restriction of the production and utilization of legacy per- and polyfluoroalkyl substances (PFAS), leading to continuous development and adoption of novel alternatives. To effectively identify the potential environmental risks from crop consumption, the levels of 25 PFAS, including fourteen perfluoroalkyl acids (PFAAs), two precursor substances and nine novel alternatives, in agricultural soils and edible parts of various crops around a fluoride industrial park (FIP) in Changshu city, China, were measured. The concentration of ΣPFAS in the edible parts of all crops ranged from 11.64 to 299.5 ng/g, with perfluorobutanoic acid (PFBA) being the dominant compound, accounting for an average of 71% of ΣPFAS. The precursor substance, N-methylperfluoro-octanesulfonamidoacetic acid (N-MeFOSAA), was detected in all crop samples. Different types of crops showed distinguishing accumulation profiles for the PFAS. Solanaceae and leafy vegetables showed higher levels of PFAS contamination, with the highest ΣPFAS concentrations reaching 190.91 and 175.29 ng/g, respectively. The highest ΣAlternative was detected in leafy vegetables at 15.21 ng/g. The levels of human exposure to PFAS through crop consumption for various aged groups were also evaluated. The maximum exposure to PFOA for urban toddlers reached 109.8% of the standard value set by the European Food Safety Authority (EFSA). In addition, short-chained PFAAs and novel alternatives may pose potential risks to human health via crop consumption.

## 1. Introduction

Per- and polyfluoroalkyl substances (PFAS), owing to their strong chemical stability, thermal stability and high surface activity, are extensively employed in various industrial sectors such as chemicals, electronics, aviation and textiles. Specifically, perfluorooctane sulfonate (PFOS) is utilized in the production of pesticides, flame retardants, textiles and in metal processing [1,2], while perfluorooctanoic acid (PFOA) serves as a processing aid (emulsifier) in the creation of fluoropolymer coatings and is added to synthetic industrial products [3]. Consequently, the ubiquitous application of PFAS results in their continuous release into the environment [4,5]. However, toxicological evidence suggests that PFAS may cause a range of detrimental effects to human health, including neurotoxicity, hepatotoxicity, carcinogenicity, reproductive and developmental toxicity, as well as immunotoxicity [6,7]. Given its potential for toxicity, bioaccumulation, persistence and long-distance migratory capacity, international attention has been further increased [8]. Legacy PFAS, such as PFOA and PFOS, have been included in international Stockholm Convention to restrict their production and use [9]. However, due to the wide range of application scenarios in industrial production attributed to their unique material properties, some short-chained PFAS and novel perfluorinated alternatives have been developed by fluorochemical manufacturers [10]. These novel alternatives have been developed to mitigate policy and regulatory uncertainties and minimize environmental risks while maintaining the excellent chemical properties of legacy PFAS such as chemical stability, hydrophobicity and lipophobicity [11]. For example, Hexafluoropropylene oxide dimmer acid (HFPO-DA) and Hexafluoropropylene oxide trimer acid (HFPO-TA) were used as alternatives to PFOA [12], while 6:2 chlorinated polyfluorinated ether sulfonate (6:2 Cl-PFESA) and 6:2 fluorotelomer sulfonate (6:2 FTS) were chosen as substitutes for PFOS. Studies have indicated that HFPO-DA can be produced at a rate of up to 100 t/a in Europe [13]. Additionally, HFPO-TA is predominantly utilized as a PFOA alternative in China, with researchers predicting total riverine emissions of HFPO homologues exceeding 8.4 t (2.4 t of HFPO-DA and 6.0 t of HFPO-TA, respectively) [14]. Moreover, 6:2 Cl-PFESA has served as an alternative to PFOS in the chromium plating industry in China for over 30 years. Monitoring of chromium plating wastewater has revealed that concentrations of the substance in the effluent and influent reached 43–78 and 65–112 μg/L, respectively [15]. It was believed that developing alternatives by shortening the length of the fluorocarbon chain or inserting heteroatoms into it (e.g., HFPO-DA), and substituting C-F bonds with C-H ones, would result in a reduced environmental impact [16]. Nevertheless, the potential toxicity of these new substitutes has been gradually revealed as research deepens [17]. Toxicological studies have indicated that the novel alternatives, e.g., HFPO-TA, may exhibit stronger hepatotoxicity compared to conventional PFAS, thereby posing greater potential health risks to humans [18,19,20]. These findings suggest that more attention should also be given to novel perfluorinated alternatives.

Crop uptake and the accumulation of PFAS in contaminated soil have been identified as crucial processes by which these substances can enter terrestrial food webs [21]. The consumption of crops contaminated with PFAS has become a significant pathway for human exposure to these chemicals [22]. Studies have confirmed the absorption and accumulation of PFAS by major food crops such as wheat, corn and soybeans, as well as vegetables like lettuce, tomatoes and radishes, in soil or nutrient solutions where PFAS are artificially introduced. Vegetable intake occupies a large proportion of the daily dietary structure of human beings; thus, contaminated crops pose potential hazards to human health [23]. However, accurately reflecting the contamination characteristics of PFAS under open-field cultivation in potting and hydroponic experiments is challenging [24,25]. Hence, it is of great significance to study field crops that have been contaminated with PFAS.

Emissions from industrial production have been identified as the main contributor to PFAS pollution [26]. In recent years, with the implementation of restrictions imposed by European and American agreements on the production and emission of PFAS, enterprises involved in the manufacture and application of PFAS have gradually moved to developing countries, including China [27]. This study was conducted in the fluoride industrial park (FIP) located in the Yangtze River Delta region of China, and the production and application of PFAS in this FIP has been carried out since 1999 [28]. Additionally, the contamination levels of PFAS in soil and different types of crops at various distances around the FIP were systematically examined. The goals of this study are: (1) to explore the occurrence characteristics, contamination and the risk of exposure to PFAS through the consumption of agricultural products grown locally; and (2) to propose planting optimization and food safety measures to reduce the risk of human exposure.

## 2. Materials and Methods

### 2.1. Sample Collection

**Figure 1:**
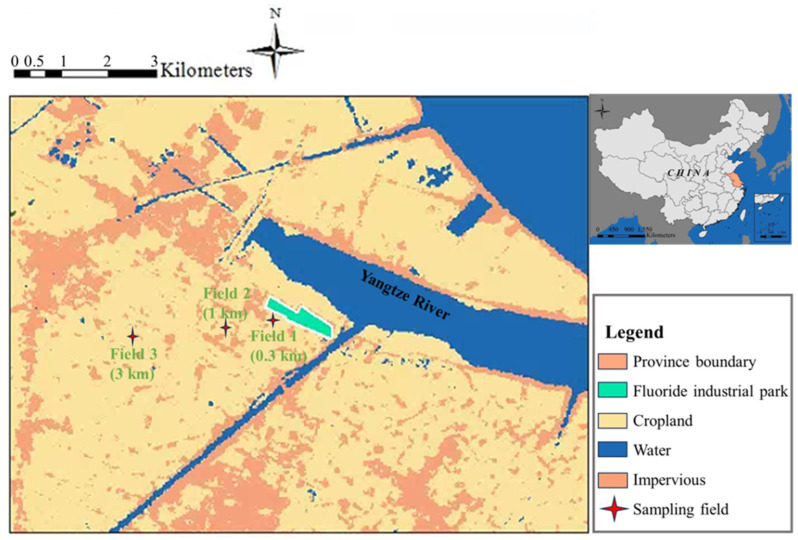
The map of the sampling locations for agricultural soil and crops around the fluoride industrial park in Changshu City, Jiangsu Province, China.

Sampling activities were conducted in August 2020 in the FIP (Changshu City, Jiangsu Province, China), an industrial park comprising various industries such as chemicals, textiles, metal processing, industrial equipment manufacturing and fluorochemicals. In addition, the area serves as an important crop production region, characterized by a dense network of rivers, extensive farmland and scattered villages. The cultivation of staple grains and various types of vegetables plays a vital role in the local diet. The sampling locations were centered around the Daikin Fluorochemical factory, starting within a 300 m radius southwest of the factory’s perimeter. Subsequently, the distance was increased to 1 km and 3 km southwest of the factory (Figure 1).

The samples collected included edible parts of various vegetables and soil samples. Specific information about the types and quantities of vegetables sampled is shown in Table S1. At each sampling point, edible parts of vegetables were randomly sampled from the center and the four corners of a 5 m × 5 m field. The vegetables of the same type were then mixed together to create a composite sample. For soil sample collection, topsoil (0–20 cm) was collected at the corresponding plant sampling points using a clean stainless steel trowel. The soil samples were also mixed to form a composite sample. After collection, all samples were placed in clean polypropylene (pp) ziplock bags and transported back to the laboratory in a −20 °C refrigerator. Once in the laboratory, crop samples were thoroughly washed with purified water, followed by Milli-Q water, and then frozen in a −40 °C refrigerator. The fully frozen samples were then freeze-dried. The freeze-dried crop samples were homogenized by grinding in a pulverizer, while the soil samples were ground using a mortar and pestle and sieved through a 2 mm mesh. To avoid cross-contamination during the grinding process, the pulverizer and mortar were rinsed and cleaned with methanol after each use. The resulting powdered samples were collected in polypropylene (pp) ziplock bags and stored in a refrigerator at −40 °C until extraction.

### 2.2. Standards and Reagents

A total of 25 PFAS were identified and quantified in all samples, including 14 PFAAs, PFBA, Perfluorohexanoic acid (PFHxA), Perfluoroheptanoic acid (PFHpA), PFOA, Perfluorononanoic acid (PFNA), Perfluorodecanoic acid (PFDA), Perfluoroundecanoic acid (PFUnDA), Perfluorododecanoic acid (PFDoDA), Perfluorobutane sulfonate (PFBS), Perfluoropentane sulfonate (PFPeS), Perfluorohexane sulfonate (PFHxS), Perfluoroheptane sulfonate (PFHpS), Perfluorooctane sulfonate (PFOS), Perfluorononane sulfonate (PFNS); 2 precursor substances, N-MeFOSAA, N-ethylperfluorooctanesulfonamidoacetic acid (N-EtFOSAA); and 9 novel fluorinated alternatives, 6:2 Cl-PFESA, 8:2 chlorinated polyfluorinated ether sulfonic acid (8:2 Cl-PFESA), Sodium 8-chloroperfluoro-1-octanesulfonate (8Cl-PFOS), HFPO-DA, HFPO-TA, Hexafluoropropylene oxide tetramer acid (HFPO-TeA), 4:2 fluorotelomer sulfonic acid (4:2 FTS), 6:2 FTS, 8:2 fluorotelomer sulfonic acid (8:2 FTS). To ensure accurate quantification, 18 mass-labelled internal standards were utilized. Detailed information on the standards and reagents used in the experiments can be found in Tables S2 and S3.

### 2.3. Sample Extraction and Instrumental Analysis

Soil and plant samples were extracted using solid-phase extraction (SPE) following the method previously described by Chen et al. [29]. Briefly, three extractions were performed on plant and soil samples using acetonitrile/water (*v*/*v* = 9:1) and acetonitrile. The extracts were then concentrated with nitrogen and underwent extraction clean-up using Oasis WAX SPE tubes as detailed in the Supplementary Materials. PFAS were quantified in negative electrospray ionization (ESI) mode using an Agilent 1290 infinity II UPLC (Agilent Technologies, Santa Clara, CA, USA) combined with an API 5500 triple-quadrupole mass spectrometer (AB SCIEX Inc., Framingham, MA, USA). Separation of the target PFAS was performed using the Waters acquity BEH C18 column (1.7 μm, 2.1 mm × 100 mm). Detailed descriptions of the instrumental analyses and mass spectral parameters for the target compounds are given in Tables S4 and S5.

### 2.4. Quality Assurance and Quality Control (QA/QC)

To avoid possible cross-contamination during sampling, soil and plant samples were individually stored in polythene ziplock bags. To monitor possible external contamination during extraction and instrumental analyses, procedure and solvent blanks were prepared using Milli-Q water and methanol, respectively. No PFAS were detected above the limit of quantification (LOQ) in all the blank samples. Concentrations over the limit of detection (LOD) were used to correct for sample concentrations. The LOD and LOQ were designated as the peaks with signal-to-noise ratios (S/N) of 3 and 10, respectively. Experiments were conducted to quantify the target analytes using 8-point matrix-matched standard curves (0.05, 0.1, 0.5, 1, 5, 10, 50 and 100 ng/mL) spiked with a 5 ng/mL internal standard. The regression coefficients (R^2^) of the calibration curves were >0.99 for all target analytes. Detailed QA/QC information can be found in Table S6.

### 2.5. Statistical Analyses and Graphic Plotting

In this study, the concentrations of PFAS in both soil and crop samples were calculated based on dry weight (dw). Statistical analysis and graphical representation were performed using SPSS Statistics V22.0 (SPSS Inc., Quarry Bay, Hongkong, China), OriginPro 9.0 (OriginLab Corporation, USA) and Excel 2016 (Microsoft Corporation, USA).

### 2.6. Dietary Intake Estimation

The consumption of contaminated crops has been identified as a significant pathway for exposure to PFAS, posing a potential risk to human health [30]. Vegetables are an essential part of the human diet, with an average intake of 121.6 kg per year, which is similar to the consumption of staple foods (126.8 kg). Therefore, it is crucial to evaluate the risk of PFAS exposure through crop intake in the daily diet. The estimated daily intake (EDI, ng/kg body weight (BW)/day) is an important indicator for assessing health risks. Therefore, the present study calculated the EDI of PFAS from different types of vegetables and cereals (maize). The calculations are given below:

(1) $$\mathrm{EDI}=\frac{\mathrm{Daliy}\;\mathrm{consumption}\;(g/d\;\mathrm{fw})\text{×}\mathrm{PFAS}\;\mathrm{concentration}\;(\mathrm{ng}/g\;\mathrm{fw})}{\mathrm{Body}\;\mathrm{weight}\;(\mathrm{kg})}$$

Data on daily consumption of the selected food items were obtained from the Exposure Factors handbook of the Chinese population (children and adults) [31,32]. The concentrations of PFAS in the edible fractions of crops were measured based on dry weight and were converted to the corresponding fresh weight (fw) based on water content. Information on the water content of different types of crops was obtained from the food nutrient content query platform of the National Institute for Nutrition and Health, Chinese Center for Disease Control and Prevention (NINH, China CDC) [33]. In order to demonstrate the most severe dietary exposure, known as the upper bound scenario, the crop with the highest moisture content was chosen for each type of crop, taking into account the large-scale production of crops and the large number of consumers in the study area. To account for variations in daily dietary intake and body weight, the EDI were calculated for three age groups, i.e., toddlers (2–5 years), children and teenagers (6–17 years) and adults (>18 years). In addition, given the sampling area’s urban–rural setting, the potential consumption of contaminated crops by urban residents was taken into account. As a result, we calculated Estimated Daily Intakes (EDIs) for different age groups in urban and rural areas separately. The specific intake and body weight data for the different crop types are presented in Table S7. Non-carcinogenic risk evaluation was performed using the hazard quotient (HQ) and hazard index (HI). An HI value exceeding 1.0 signifies a high risk level, whereas an HI value below 1.0 indicates a low risk level [34].

(2) HQ=EDI∕RfD

(3) HI=∑HQ

The RfD values for PFBA and PFBS were 2900 ng/kg BW/d and 430 ng/kg BW/d, respectively, and were sourced from the Minnesota Department of Health (MDH) [35]. A RfD value of 320 ng/kg BW/d for PFHxA was obtained from the French Agency for the Safety of Food, Environment and Occupational Health and Safety (ANSES) [36]. According to the EPA report, the RfD values for HFPO-DA and PFOA were 30 ng/kg BW/d and 20 ng/kg BW/d, respectively [37,38].

## 3. Results and Discussion

### 3.1. PFAS in Agricultural Soils

**Figure 2:**
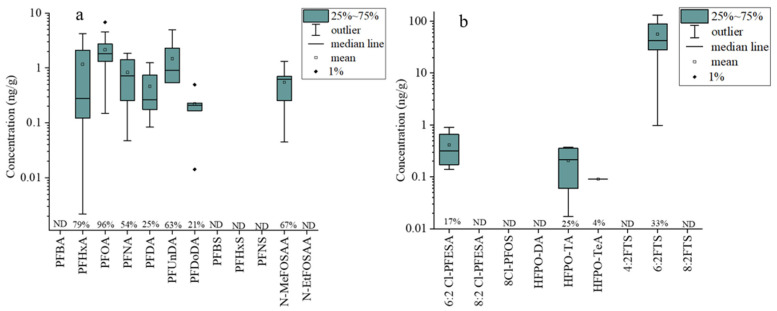
The concentrations of different PFAAs and precursor compounds (**a**), as well as novel alternatives (**b**), in agricultural soils. Percentage at the bottom of the diagram represents the detection frequency (%). ND, nondetectable.

A total of 20 different PFAS were detected in the soil samples. The highest concentration of the total PFAS (∑PFAS) detected in soil samples was 133.13 ng/g. The levels of total PFAAs (∑PFAAs) in the soil samples ranged from 0.15 to 13.98 ng/g, while those of total novel alternatives (∑Alternatives) ranged from 0.05 to 131.1 ng/g. Among the precursors, N-MeFOSAA was detected with a detection rate of 67% and an average concentration of 0.55 ng/g (Figure 2a). PFOA was also commonly detected in 96% of the soils, with a maximum concentration of 6.8 ng/g (Figure 2a). Listed as a persistent organic pollutant (POP) in the Stockholm Convention in 2019 [39], PFOA has a high detection rate and concentration due to its previous extensive use and strong hydrophobicity, which causes it to readily adsorb to soil particles and limits its ability to migrate through soil systems [40]. In a related study on PFAS contamination in Uganda, the highest PFAS level detected in wetlands and surrounding agricultural soils (∑PFAS = 3000 pg/g) was only 21.1% of the highest soil ∑PFAAs found in this study [41]. The detection rate of novel fluorinated alternatives in the soil was relatively low, with the highest detection rate observed for 6:2 FTS at 33% with an average concentration of 56.37 ng/g (Figure 2b). In a study of surface water and sediment around the FIP (Changshu), the concentrations of 6:2 FTS in the surface water and sediment ranged from 10.3 to 402 ng/L and 32.8 to 46.1 ng/g, respectively [42], which were similar to the concentrations detected in the soil in this study.

### 3.2. PFAS in Edible Parts of Crops

**Figure 3:**
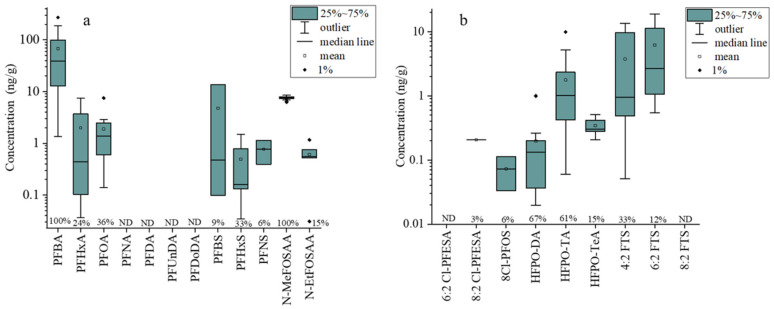
The concentrations of different PFAAs and precursor compounds (**a**), as well as novel alternatives (**b**), in edible parts of crops. Percentage at the bottom of the diagram represents the detection frequency (%). ND, nondetectable.

The concentrations of PFAS in the edible parts of crops were determined. The highest concentrations of the ∑PFAS detected in crop samples were 299 ng/g. The levels of ∑PFAAs in the crop samples were 2.35–286.64 ng/g, while those of ∑Alternatives were 0.03–19.10 ng/g. PFBA was detected in all of the crop samples, with a maximum concentration of 272 ng/g (Figure 3a), accounting for 84% of the ∑PFAS content in all of the crop samples. Short-chained PFAS, due to their smaller molecular size and higher water solubility, are more likely to pass through root cell walls and membranes, enter the xylem tissues of plants through water transpiration and then translocate to the shoot, where they are more likely to be taken up and accumulated by the plants compared to the long-chained PFAS [43]. While previous studies have shown that PFOA tends to accumulate in the roots of crops [44], the detection rate of PFOA in edible parts of the crops in this study was 36%, with an average concentration of 1.9 ng/g (Figure 3a). A previous study in Saudi Arabia reported the highest concentration of ∑PFAAs in agricultural crops to be 90.2 ng/g, with an average concentration of 20.5 ng/g. The contamination of agricultural crops in this study exceeded this level, with emissions from the FIP contributing to the increased PFAS contamination in the study area [45]. It is worth noting that, although the precursor compound N-MeFOSAA did not contribute significantly to the cumulative concentration of PFAS in crops, it was detected in 100% of crops with an average concentration of 7.6 ng/g (Figure 3a). Fewer toxicological studies have been conducted on precursor substances; however, a previous study showed that N-MeFOSAA has a high detection rate in cerebrospinal fluid and may pose a potential risk for the development of behavioral disorders and cognitive developmental deficits in humans [46]. Among the novel alternatives of concern in the study, hexafluoropropylene oxide (HFPO) homologues were detected in 73% of all of the alternatives, with an average concentration of 0.88 ng/g (Figure 3b). The highest detected concentration among the alternatives was 6:2 FTS, with an average concentration of 6.18 ng/g (Figure 3b). A previous study indicated that 6:2 FTS undergoes a biotransformation in plants, resulting in the formation of metabolites such as PFHpA, PFHxA, PFBA, Perfluoropentanoic acid (PFPeA), PFBA and Perfluoropropionic acid (PFPrA). These metabolites can pose significant health risks through the food chain [47].

### 3.3. Spatial Distribution of PFAS around the FIP

**Figure 4:**
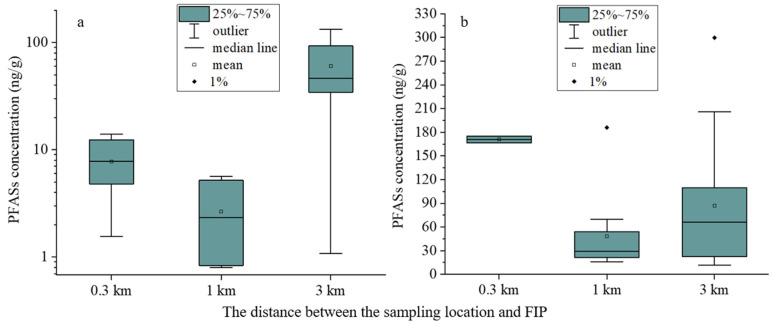
Spatial distributions of ΣPFAS in agricultural soils (**a**) and edible parts of crops (**b**) in the area around the fluoride industrial park.

The concentration of PFAS in the soil varied depending on the distance from the FIP. The higher concentration of PFAS at the 3 km sampling site, compared to the 1 km site, was primarily due to the elevated levels of 6:2 FTS in the soil at the 3 km site, where it accounted for more than 90% of the PFAS present (Figure 4a and Figure S1). The 6:2 FTS alternative is extensively used in the electroplating industry, fire-fighting foams and as a surface treatment agent for industrial products like leather, textiles and wood [48]. A previous study on the distribution and potential release of PFAS in the fire training site revealed high levels of contamination, with 6:2 FTS concentrations in the concrete ranging from 23 to 553 ng/g [49]. The 3 km sampling area of this study is in close proximity to several apparel and electroplating companies, as well as the FIP’s fire station. These various potential emission sources may have contributed to the elevated concentrations of 6:2 FTS detected in this area.

The Daikin Fluorochemicals factory is one of the largest and most productive companies in the area [23], and crops within 300 m of the plant show the highest concentrations of ΣPFAS (Figure 4b). There was a general trend of decreasing levels of PFAS detected in the crops as the distance increased. However, the concentration of PFAS was higher in the crops at the 3 km location compared to the 1 km location (Figure 4b). The main reason for this phenomenon could be attributed to the presence of numerous small- and medium-sized fluoride factories to the north-east of the 3 km sampling area. These factories potentially impact the concentration levels of PFAS in crops through atmospheric precipitation [50]. Additionally, the sampling site is located near a tributary of the Fushan Pond, where several small- and medium-sized fluoride factories are located upstream. The use of this water source for irrigation by local residents may further contribute to the contamination of soil and crops with PFAS [51].

### 3.4. Contamination of PFAS in Different Crop Species

**Figure 5:**
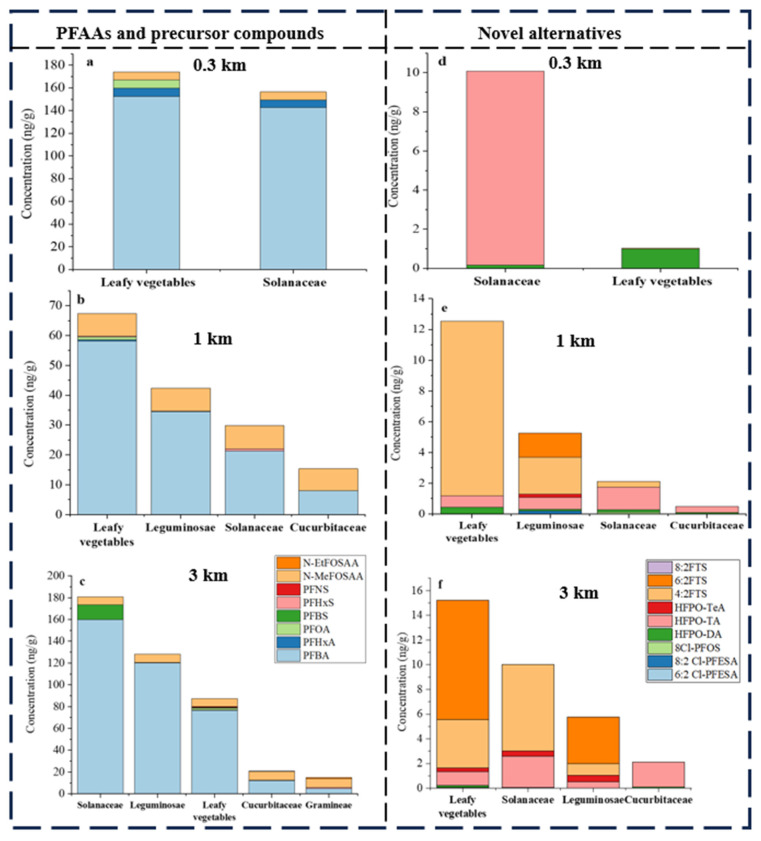
The concentration of PFAAs, precursor compounds and novel alternatives in different crops collected from various distances from FIP (0.3, 1, and 3 km). The bar graphs display the average concentration.

Various types of crops, such as leafy vegetables, solanaceae, cucurbitaceae, gramineae and leguminosae, were gathered in the sampling area, representing a significant portion of the local population’s daily diet. The specific crop species collected are detailed in Table S1. The composition of crops collected at different distances varied, with leafy vegetables and solanaceae being present at all three sampling locations (0.3, 1, and 3 km). PFBA was identified as contributing to over 50% of the contamination load of ∑PFAS in the crops across all sampling sites, except for gramineae crops from the 3 km location (Figure 5). At the 0.3 km site, both leafy vegetables and solanaceae exhibited similar ∑PFAAs concentrations of 167.3 and 149.5 ng/g, respectively, with PFBA accounting for a significant portion of the ∑PFAAs load in both crops (Figure 5a). At the 1 km area, leafy vegetables had the highest concentration of ∑PFAAs at 59.7 ng/g, while solanaceae showed a 63% lower concentration compared to leafy vegetables, with PFBA again playing a major role in the contamination (Figure 5b). In contrast, the highest ∑PFAAs concentration in solanaceae at 173.5 ng/g was found at the 3 km location, with PFBA contributing to a large portion of the contamination load in all crop types (Figure 5c). Overall, leafy vegetables absorbed and accumulated the most PFAAs, including long-chained ones like PFOA, PFHxS and PFNS. Legacy PFAS like PFOA were detected in leafy vegetables at various sampling points, with the highest mean concentration observed at the 0.3 km site (Figure 5a–c). In comparison to the other crop types, gramineae and cucurbitaceae crops exhibited lower average concentrations of legacy PFAS. The highest concentration of PFBA, a specific substance, was found in cucurbitaceae at 3 km area with an average concentration of 12.01 ng/g (Figure 5c). Gramineae samples, on the other hand, were only collected at the 3 km location, where PFBA was also detected at an average concentration of 4.8 ng/g (Figure 5c). N-MeFOSAA, a precursor substance, showed similar accumulation levels in various crop types across different sampling sites, with average concentrations of 7.72 and 8.25 ng/g even in the cucurbitaceae and gramineae from the 3 km site, where the accumulation of PFAAs was low (Figure 5a–c).

Analyses of the concentrations of the novel alternatives in various crops demonstrated that leafy vegetables consistently exhibited high levels of enrichment at both the 1 and 3 km sites (Figure 5e,f). The main alternatives identified were 6:2 FTS and 4:2 FTS, with an average concentration of 11.34 ng/g of 4:2 FTS in leafy vegetables, representing 90.4% of the leafy vegetable ∑Alternatives load at the 1 km location (Figure 5e). In the 3 km area, the highest average concentration was found to be 9.67 ng/g of 6:2 FTS, accounting for 63.6% of the ∑Alternatives load (Figure 5f). These findings can be attributed not only to the fugitive behavior of pollutants in the study area but also to the specific uptake and accumulation characteristics of certain crop species. A study on the uptake and accumulation characteristics of PFAS in cabbage revealed that 6:2 FTS exhibited a higher bioaccumulation in cabbage leaves, with higher transfer factors (TFs) from roots to stems and from stems to leaves, indicating that substitutes developed by replacing fluorine atoms with hydrogen atoms have greater mobility and are more readily transported to the shoot parts, thereby increasing the risk of food contamination [52]. HFPO homologues were also commonly detected in various crops, with high mean concentrations of HFPO-TA in solanaceae across the different sampling sites (Figure 5d–f). The highest mean concentration of 10 ng/g was observed at the 0.3 km site, constituting 98.3%, 69.3% and 25.5% of the ∑Alternatives loadings in solanaceae at the three sampling sites, respectively (Figure 5d–f). In contrast, the accumulation of alternatives in cucurbitaceae differed significantly from the other crop types, with only HFPO homologues being detected (HFPO-DA: 0.084 ng/g, 0.078 ng/g; HFPO-TA: 0.41 ng/g, 2.04 ng/g, Figure 5e,f).

Different types of crops showed varying characteristics of PFAS accumulation. Solanaceae, leafy vegetables and leguminosae demonstrate higher accumulation levels. Leafy vegetables, with a significant proportion of edible parts, may experience a higher level of PFAS accumulation due to increased transpiration flows and a larger leaf area, making them more susceptible to the airborne deposition of PFAS [53]. The higher levels of accumulation in leguminosae, on the other hand, can be attributed to their higher protein and lipid content, which enhances the adsorption and bioaccumulation of PFAS [54]. In the present study, the lower amount of accumulation of PFAS in the edible part of the gramineae (maize) may be attributed to the stronger biological barrier between the root and seed in maize, the longer transit distance within the plant and the lower protein and lipid content of maize kernels [23]. Conversely, for cucurbitaceae, it has been found that the leaves play a crucial role in the accumulation of PFAS, which could explain the lower levels of PFAS in the edible parts [25]. It is important to note that sampling was conducted in August, one of the most critical growing seasons of the year in the study area and a suitable time for our contamination investigation. However, future studies should focus on examining soil levels before, during and after the growing season.

### 3.5. Assessment of the Health Risks to the Local Population Associated with Exposure to PFAS

In this study, the highest detected concentrations of PFOA and 6:2 FTS in soils near the FIP were 6.79 and 131.1 ng/g, respectively, which were much lower than the current predicted no-effect concentrations of PFOA (160 ng/g) and 6:2 FTS (210 ng/g) [55]. However, considering the significant contribution of crops to the human daily dietary intake, the potential risk to human health from enriched PFAS cannot be ignored [56]. Therefore, this study aimed to calculate the EDI values of PFAS consumed through crops in the daily diets of urban and rural residents across different age groups. In addition, it aimed to investigate the risk of PFAS exposure resulting from the intake of different types of crops by the residents (Table 1).

PFBA accounted for the majority of PFAAs intake, with the highest concentration at 50.11 ng/kg BW/d, followed by PFBS (4.83 ng/kg BW/d) and PFHxS (1.88 ng/kg BW/d). These pollutants accounted for 84.2–99.68%, 0.33–11.94% and 0.14–15.8% of the EDI load of ∑PFAAs, respectively. Calculations suggest that toddlers, children and teenagers have a higher intake of PFAAs compared to adults, which is possibly due to the relatively homogeneous diets of toddlers, children and teenagers, and their higher food consumption per kg of BW. Additionally, adults living in rural areas have a higher projected intake of PFAAs compared to those in urban areas. This could be attributed to the higher intake of vegetables in rural areas compared to urban areas [57]. For the precursors, N-MeFOSAA is of particular interest, with an EDI range of 1.13–20.05 ng/kg BW/d and a mean concentration of 4.56 ng/kg BW/d. Residents of all age groups were more highly exposed to N-MeFOSAA compared to the PFAAs, except for PFBA. Regarding the novel alternatives studied, 6:2 FTS had higher EDI values compared to 4:2 FTS (0.94–3.25 ng/kg BW/d, 0.59–1.55 ng/kg BW/d), followed by HFPO-TA (0.22–0.68 ng/kg BW/d). These alternatives accounted for 47.35–56.91%, 27.18–57.22% and 11.93–94.89% of the ΣEDI, respectively.

The results of ΣEDI for PFAAs ingested through different types of crops showed leguminosae > solanaceae > leafy vegetables > cucurbitaceae. The concentrations of PFAAs in the edible parts of solanaceae, leguminosae and leafy vegetables were similar, and the differences in ΣEDI were mainly due to the variations in water content among the different types of crops. Leguminosae had increased exposure due to their lower water content. The highest EDI values for precursor compounds, particularly N-MeFOSAA, were found in gramineae. In the urban toddler group, the EDI value reached 20.05 ng/kg BW/d, and the mean value across different groups was 14.24 ng/kg BW/d. However, it is important to note that this study only sampled a single crop, maize, from the gramineae family due to time and location constraints. Therefore, the exposure of the local population to PFAS through the consumption of gramineae may not be fully reflected, as rice is the predominant form of gramineae intake in their daily diet. Leafy vegetables, compared to other crop types, had high EDI values for the remaining PFAS studied, except for the precursor compounds. The ΣEDI of leafy vegetables for the alternatives reached 23.98 ng/kg BW/d, with 6:2 FTS contributing the most (57%) with an average EDI value of 2.275 ng/kg BW/d. The ΣEDI values of the other types of crops for the alternatives ranged from 1.8 to 15.42 ng/kg BW/d, excluding gramineae.

Among the PFAS of concern, PFBA was found to be the contaminant with the highest risk of exposure to the local population through the consumption of crops. The maximum intake of PFBA (50.11 ng/kg BW/d) was much lower than the reference dose (RfD) recommended by the Minnesota Department of Health (2900 ng/kg BW/d) [35]. The calculated HI values for PFBA, PFHxA, PFOA, PFBS and HFPO-DA were consistently below one across all crop types in this study. The estimated range of HI values for consumption of contaminated crops by urban and rural dwellers at various age points was 0.002–0.051 (Table S8), indicating a low non-carcinogenic risk associated with consuming these crops. However, for the other PFAS of concern in our research (e.g., PFBS, N-MeFOSAA, and 6:2 FTS), it was not possible to provide clarification due to the lack of corresponding assessment thresholds. To our knowledge, tolerable daily intake (TDI) values for health risk assessment currently exist only for PFOA and PFOS. In this study, the highest EDI value of PFOA in the upper bound scenario was 0.69 ng/kg BW/d, which is far lower than the current TDI value (1500 ng/kg BW/d). However, the maximum estimated weekly intake (EWI, seven times EDI) of PFOA for urban toddlers (4.83 ng/kg BW/week) exceeded the tolerable weekly intake (TWI) established by the European Food Safety Authority (EFSA, 4.4 ng/kg BW/week) [58]. The alternatives have similar structures and modes of action to PFOA and PFOS, and generally exhibit toxic effects including hepatotoxicity and immunotoxicity. With in-depth studies on human and ecotoxicology, future TDI values may incorporate the risk to human health from co-exposure to these substances. It is worth noting that the production scale of novel alternatives has been expanding without restriction. Local populations are exposed to increased health risks from continued exposure to these substances due to the lack of safer alternatives or effective control policies. To minimize the health risk associated with crop consumption, it is recommended to cultivate crop varieties with low PFAS accumulation instead of solanaceae and leafy vegetables near the FIP. Additionally, there is a potential risk of exposure for humans through the use of leafy vegetables as animal feed, which should be monitored in the future.

## 4. Conclusions and Environmental Implications

Considering the continuous movement of PFAS-related industries to agriculture-intensive developing countries and the growing production and use of novel alternatives, this study investigated the distribution and occurrence of PFAS in the industrial–agricultural interaction and evaluated the associated health risks. The concentrations of PFAS in various types of soil and edible parts of crop samples were explored. The results of this study revealed that the concentrations of ΣPFAS in the edible parts of all crops ranged from 11.64 to 299.5 ng/g, with PFBA being the dominant compound, accounting for an average of 71% of the ΣPFAS. Among the crops, solanaceae showed the highest levels of ΣPFAS (maximum 190.91 ng/g), while cereals exhibited the lowest (14.77 ng/g). Leafy vegetables had the highest contents of ΣAlternative (maximum 15.21 ng/g). The precursor compound N-MeFOSAA was detected in all of the crop samples, with an average concentration of 7.6 ng/g. The risk of human exposure to PFAS through the consumption of different types of crops was evaluated using established criteria such as RfD, TDI and TWI values. The estimated PFOA exposure of urban toddlers through crop consumption accounted for 109.8% of the TWI value set by the EFSA, suggesting that specific local populations may face potential health risks from consuming crops. However, the lack of appropriate assessment criteria for the PFAS we were concerned with in this study hindered a more accurate risk assessment. Obtaining additional toxicological data and exposure hazard information on PFAS, especially novel alternatives, is crucial for establishing more comprehensive criteria for risk assessment.

## Figures and Tables

**Table 1:** Estimated daily intake (EDI, ng/kg BW/d) values of different types of crops.

EDIs			EDI (Cucurbitaceae)														EDI (Leguminosae)													
Age			2–5 y				6–17 y					>18 y					2–5 y					6–17 y					>18 y			
Area			U		R		U			R		U			R		U		R			U		R			U		R	
PFBA			3.05		2.71		1.79			1.85		1.65			1.76		50.11		44.57			29.42		30.42			27.18		28.92	
PFHxA			ND		ND		ND			ND		ND			ND		0.07		0.06			0.04		0.04			0.04		0.04	
PFOA			0.04		0.03		0.02			0.02		0.02			0.02		ND		ND			ND		ND			ND		ND	
PFBS			ND		ND		ND			ND		ND			ND		ND		ND			ND		ND			ND		ND	
PFHxS			0.13		0.11		0.07			0.08		0.07			0.07		0.09		0.08			0.05		0.06			0.05		0.05	
PFNS			ND		ND		ND			ND		ND			ND		ND		ND			ND		ND			ND		ND	
N-MeFOSAA			2.08		1.85		1.22			1.26		1.13			1.20		5.02		4.47			2.95		3.05			2.73		2.90	
N-EtFOSAA			0.15		0.14		0.09			0.09		0.08			0.09		0.02		0.02			0.01		0.01			0.01		0.01	
8:2 Cl-PFESA			ND		ND		ND			ND		ND			ND		0.13		0.12			0.08		0.08			0.07		0.08	
8Cl-PFOS			ND		ND		ND			ND		ND			ND		ND		ND			ND		ND			ND		ND	
HFPO-DA			0.02		0.02		0.01			0.01		0.01			0.01		0.04		0.04			0.02		0.03			0.02		0.02	
HFPO-TA			0.41		0.36		0.24			0.25		0.22			0.23		0.44		0.39			0.26		0.27			0.24		0.26	
HFPO-TeA			ND		ND		ND			ND		ND			ND		0.23		0.21			0.14		0.14			0.13		0.13	
4:2 FTS			ND		ND		ND			ND		ND			ND		1.08		0.96			0.64		0.66			0.59		0.62	
6:2 FTS			ND		ND		ND			ND		ND			ND		1.74		1.55			1.02		1.05			0.94		1.00	
**EDIs**	**EDI (Leafy Vegetables)**										**EDI (Solanaceae)**										**EDI (Gramineae)**									
**Age**	**2–5 y**			**6–17 y**				**>18 y**			**2–5 y**			**6–17 y**				**>18 y**			**2–5 y**				**6–17 y**			**>18 y**		
**Area**	**U**	**R**		**U**		**R**		**U**	**R**		**U**		**R**	**U**		**R**		**U**		**R**	**U**		**R**		**U**	**R**		**U**		**R**
PFBA	27.33	24.32		16.05		16.59		14.82	15.78		35.32		31.42	20.74		21.44		19.15		20.38	11.66		9.79		10.02	6.68		5.15		6.35
PFHxA	0.88	0.78		0.52		0.53		0.48	0.51		ND		ND	ND		ND		ND		ND	ND		ND		ND	ND		ND		ND
PFOA	0.69	0.61		0.40		0.42		0.37	0.40		ND		ND	ND		ND		ND		ND	ND		ND		ND	ND		ND		ND
PFBS	0.10	0.09		0.06		0.06		0.05	0.06		4.83		4.29	2.83		2.93		2.62		2.79	ND		ND		ND	ND		ND		ND
PFHxS	0.04	0.04		0.02		0.02		0.02	0.02		0.28		0.25	0.16		0.17		0.15		0.16	2.19		1.84		1.88	1.25		0.97		1.19
PFNS	0.26	0.23		0.15		0.16		0.14	0.15		ND		ND	ND		ND		ND		ND	ND		ND		ND	ND		ND		ND
N-MeFOSAA	2.47	2.19		1.45		1.50		1.34	1.42		2.66		2.36	1.56		1.61		1.44		1.53	20.05		16.84		17.24	11.49		8.86		10.93
N-EtFOSAA	ND	ND		ND		ND		ND	ND		ND		ND	ND		ND		ND		ND	1.98		1.67		1.71	1.14		0.88		1.08
8:2 Cl-PFESA	ND	ND		ND		ND		ND	ND		ND		ND	ND		ND		ND		ND	ND		ND		ND	ND		ND		ND
8Cl-PFOS	0.01	0.01		0.01		0.01		0.01	0.01		0.04		0.04	0.02		0.02		0.02		0.02	ND		ND		ND	ND		ND		ND
HFPO-DA	0.12	0.10		0.07		0.07		0.06	0.07		0.04		0.03	0.02		0.02		0.02		0.02	ND		ND		ND	ND		ND		ND
HFPO-TA	0.68	0.61		0.40		0.41		0.37	0.39		0.74		0.66	0.44		0.45		0.40		0.43	ND		ND		ND	ND		ND		ND
HFPO-TeA	0.10	0.09		0.06		0.06		0.05	0.06		0.15		0.13	0.09		0.09		0.08		0.08	ND		ND		ND	ND		ND		ND
4:2 FTS	1.55	1.38		0.91		0.94		0.84	0.90		1.29		1.15	0.76		0.79		0.70		0.75	ND		ND		ND	ND		ND		ND
6:2 FTS	3.25	2.89		1.91		1.97		1.76	1.87		ND		ND	ND		ND		ND		ND	ND		ND		ND	ND		ND		ND

Note: U, urban areas; R, rural areas; ND, nondetectable; 2–5 y represents toddlers, 6–17 y represents children and teenagers, >18 y represents adults.

## Data Availability

Data are contained within the article and Supplementary Materials.

## Supplementary Materials

The following supporting information can be downloaded at: https://www.mdpi.com/article/10.3390/toxics12040269/s1, Table S1: Sampling information for crops; Table S2: Information on reagents and materials used in the experiment; Table S3: The particular chemical names and chemical formula of the target analytes; Table S4: UPLC-MS/MS instrument parameters for the quantification of the target PFAS; Table S5: Optimal UPLC-MS/MS parameters for the target PFAS and internal standards; Table S6: The matrix spike recoveries (MSRs ± SD) of individual PFAS in crops and soil; Table S7: Reference daily crop intake (g/d), body weight (BW, kg) and maximum moisture content of different crops for three age groups in urban and rural areas of China; Table S8: HR from consumption of contaminated crops by urban and rural residents of different age groups; Figure S1: Proportion of different PFAS in soil and crop samples.
