# Supplementary material for: Crop Contamination and Human Exposure to Per- and Polyfluoroalkyl Substances around a Fluorochemical Industrial Park in China
Source: Toxics. 2024 Apr 4;12(4):269. doi: 10.3390/toxics12040269 (PMC11054258; doi:10.3390/toxics12040269)
Supplement: Supplementary file 1 [file toxics-12-00269-s001.zip › toxics-2891869-supplementary.pdf]

Supporting Information for

Crop Contamination and Human Exposure to Per- and Polyfluoroalkyl Substances around a Fluorochemical Industrial Park in China

Kairan Xu [1], Jian Huang [1], Yufeng Zhang [1], Xilong Wu [1], Dan Cai [2,3,*], Guocheng Hu [2,3], Yu Li [2,4], Zhuobiao Ni [1], Qingqi Lin [1,*], Shizhong Wang [5,6] and Rongliang Qiu [1,5]

Maoming and Heyuan Branch, Guangdong Laboratory for Lingnan Modern Agriculture, Guangdong
Provincial Key Laboratory of Agricultural & Rural Pollution Abatement and Environmental Safety, College of Natural Resources and Environment, South China Agricultural University, Guangzhou 510642, China; xukairan@stu.scau.edu.cn (K.X.); huangjian_scau@foxmail.com (J.H.); zhangyufeng1214@foxmail.com (Y.Z.); caramel_ln@foxmail.com (X.W.); nizhuobiao@scau.edu.cn (Z.N.); qiurl@scau.edu.cn (R.Q.)

2 South China Institute of Environmental Sciences, Ministry of Ecology and Environment,
Guangzhou 510655, China;

3 State Environmental Protection Key Laboratory of Environmental Pollution Health Risk Assessment, Guangzhou 510655, China

4 State Environmental Protection Key Laboratory of Urban Ecological Simulation and Protection,
Guangzhou 510655, China

5 School of Environmental Science and Engineering, Sun Yat-sen University, Guangzhou 510006, China;

6 Guangdong Provincial Key Laboratory of Environmental Pollution Control and Remediation Technology, Guangdong Provincial Engineering Research Center for Heavy Metal Contaminated Soil Remediation, Guangzhou 510006, China

* Correspondence: caidan@scies.org (D.C.); linqingqi@scau.edu.cn (Q.L.)

Includes:

16 Pages

1 Supplementary M

8 Tables

1 Figure

Supplementary M

Standards and Reagents

The 14 PFAAs, 2 precursors and 9 novel alternatives used in the experiments were purchased from Wellington Laboratories with purities of >98% (Guelph, Ontario, Canada) (Table S3). Information on other reagents used in the experiment is given in Table S2.

Sample extraction

The extraction methods for soil and plant samples were based on published methods with minor modifications and optimisation. For the extraction of soil samples, 0.5 g of freeze-dried and ground soil was placed in a 50 mL polypropylene centrifuge tube (PP), to which 50 μL of internal standard mix (100 ng/mL) was added. Add 5 ml of acetonitrile/water (90:10, v/v), vortex for 60 s and sonicate in a water bath for 10 min. Centrifuge the sonicated sample at 8000 rmp for 10 min and transfer the supernatant to another 50 mL polypropylene centrifuge tube. The supernatant was transferred to another 50 mL pp tube. 5 mL of acetonitrile was added to the soil sample tube and the vortexing and centrifugation steps were repeated to collect the supernatant. Finally, 5 mL of acetonitrile/water was added and the above vortexing and centrifugation steps were repeated, and the supernatant was collected in the above 50 mL centrifuge tube (total volume of about 15 mL). The collected supernatant was concentrated to approximately 0.5 mL using nitrogen at 40 °C. Experiments were carried out using OASIS WAX-SPE to purify the target

extract. The WAX cartridges were first pretreated with 5 mL of methanol and 5 mL of Milli-Q water was added after the methanol flow-through. The remaining 0.5 mL of the nitrogen purge was added to the WAX cartridges using a polypropylene disposable burette. 1 mL of methanol was added to the centrifuge tube and the burette was cleaned by ultrasonication for 1 min, then the methanol was added to the WAX cartridges. 10 mL of Milli-Q water was added to the centrifuge tube, and the ultrasonication step was repeated for 1 min, and the cleaned water was added to the WAX cartridges. The water in the tube was cleaned and added to the WAX cartridges. After the liquid in the WAX cartridges was completely dripped, vacuum pump was used to evacuate for 1 h. The eluate was collected using a 10 mL polyethylene centrifuge tube, 4 mL of methanol was added to the WAX cartridges first, and then 4 mL of 0.1% ammonia hydroxide methanol (ammonia hydroxide/methanol, v/v) was added after the drop was completed. The collected eluent was concentrated at 40 °C until nearly dry, then 1mL of methanol was added to the nitrogen-blown 10mL centrifuge tube to be fixed, vortexed for 60 s and then ultrasonicated for 10 min. 1mL syringe was used to absorb the fixed solution, and then the filter head was pumped into a 2mL injection bottle, which was placed in the refrigerator at -20 °C for storage until the test was performed on the machine.

Plant samples were extracted in the same way as the soil samples described above, with the main difference in the procedure being the use of organic solvents to extract the PFASs from the samples. Since it was difficult to

centrifuge the lyophilised and ground plant tissues, we used filter paper to wrap the plant powder in a 50 mL PP centrifuge tube, and then extracted it in a similar way to the soil sample extraction described above. The contaminants were extracted by adding acetonitrile and acetonitrile/water (90:10, v/v) for ultrasonic cleaning, respectively. The organic solvents were added to submerge the filter paper, and the amount of solution added was about 14 mL. The filter paper used in the experiment was cut to the appropriate size and washed three times with methanol to avoid possible contamination with PFASs. The solution obtained from the three extractions was collected (about 42 mL) and concentrated by nitrogen blowing. Unlike soil samples, plant samples, due to the presence of chlorophyll, required the addition of 0.025 g of ENVI-Carb to the WAX cartridges prior to its activation. Other than that, the rest of the purification steps were the same as for the soil samples. The prepared samples were stored in a refrigerator at -20 °C to be tested on the machine.

Table S1. Sampling information for crops.

Classification	Samples name	Number of samples	Type of samples
Central area within 300 m from the FIP	Sweet potato leaf	n=1	Leafy vegetables
	Pepper	n=1	Solanaceae
1 km from the FIP	Cabbages	n=1	Leafy vegetables
	Water spinach	n=1	Leafy vegetables
	Chinese chive	n=1	Leafy vegetables
	Scallion	n=1	Leafy vegetables
	Eggplant	n=1	Solanaceae
	Bell pepper	n=1	Solanaceae
	Pepper	n=1	Solanaceae
	Sponge gourd	n=1	Cucurbitaceae
	French bean	n=1	Leguminosae
	Snow pea	n=1	Leguminosae
3 km from the FIP	Sweet potato leaf	n=2	Leafy vegetables
	Water spinach	n=1	Leafy vegetables
	Celery	n=1	Leafy vegetables

Spinach	n=1	Leafy vegetables
Chinese chive	n=2	Leafy vegetables
Scallion	n=1	Leafy vegetables
Eggplant	n=3	Solanaceae
Pepper	n=1	Solanaceae
Maize	n=3	Gramineae
Sponge gourd	n=2	Cucurbitaceae
Cucumber	n=2	Cucurbitaceae
French bean	n=1	Leguminosae
Soybean	n=1	Leguminosae

Table S2. Information on reagents and materials used in the experiment

Reagents	Purity /Specification	Manufacturer
Methanol	HPLC-grade	Anpel[a]
Acetonitrile	HPLC-grade	Anpel
Ammonium acetate	>99%	Aladdin[b]
Aqueous ammonia	>99%	Aladdin
Supelclean graphitized carbon (ENVI-Carb)	HPLC-grade	Supelco[c]
Oasis WAX SPE cartridges	6 cc, 500 mg	Waters[d]
GHP Pall membrane	13 mm, 0.2 μm	Pall Corp[e]

[a]Anpel, Anpel Laboratory Technologies Inc (Shanghai, China);

[b]Aladdin, Aladdin Testing Co. Ltd. (Shanghai, China);

[c]Supelco, Supelco (Bellefonte, PA, USA);

[d]Waters, Waters Corporation (Milford, MA, USA);

[e]Pall Corp, Pall Corp (Port Washington, NY, USA).

Table S3. The particular chemical names and chemical formula of the target analytes.

Abbreviation	Chemical name	Chemical formula
**Perfluoroalkyl carboxylic acids PFCAs**		
PFBA	Perfluorobutanoic acid	$C_4HF_7O_2$
PFHxA	Perfluorohexanoic acid	$C_6HF_{11}O_2$
PFHpA	Perfluoroheptanoic acid	$C_7HF_{13}O_2$
PFOA	Perfluorooctanoic acid	$C_8HF_{15}O_2$
PFNA	Perfluorononanoic acid	$C_9HF_{17}O_2$
PFDA	Perfluorodecanoic acid	$C_{10}HF_{19}O_2$
PFUnDA	Perfluoroundecanoic acid	$C_{11}HF_{21}O_2$
PFDoDA	Perfluorododecanoic acid	$C_{12}HF_{23}O_2$
**Perfluoroalkane sulfonic acids PFSAs**		

PFBS	Perfluorobutane sulfonate	$C_4F_9SO_3H$
PFPeS	Perfluoropentane sulfonate	$C_5F_{11}SO_3H$
PFHxS	Perfluorohexane sulfonate	$C_6F_{13}SO_3H$
PFHpS	Perfluoroheptane sulfonate	$C_7F_{15}SO_3H$
PFOS	Perfluorooctane sulfonate	$C_8F_{17}SO_3H$
PFNS	Perfluorononane sulfonate	$C_9F_{19}SO_3H$
**Precursor substances**		
N-MeFOSAA	N-methylperfluorooctanesulfonamidoacetic acid	$C_{11}H_6F_{17}NO_4S$
N-EtFOSAA	N-ethylperfluorooctanesulfonamidoacetic acid	$C_{12}H_8F_{17}NO_4S$
**Alternatives**		
8Cl-PFOS	Perfluoro-8-chloro-1-octanesulfonic acid	$C_8HClF_{16}O_3S$
6:2 Cl-PFESA	6:2 chlorinated polyfluorinated ether sulfonic acid	$C_8HClF_{16}O_4S$

8:2 Cl- PFESA	8:2 chlorinated polyfluorinated ether sulfonic acid	$C_{10}HClF_{20}O_4S$
HFPO-DA	Hexafluoropropylene oxide dimer acid	$C_6HF_{11}O_3$
HFPO-TA	Hexafluoropropylene oxide trimer acid	$C_9HF_{17}O_4$
HFPO-TeA	Hexafluoropropylene oxide tetramer acid	$C_{12}HF_{23}O_5$
4:2 FTS	4:2 fluorotelomer sulfonic acid	$C_6H_5F_9O_3S$
6:2 FTS	6:2 fluorotelomer sulfonic acid	$C_8H_5F_{13}O_3S$
8:2 FTS	8:2 fluorotelomer sulfonic acid	$C_{10}H_5F_{17}O_3S$
**Internal standards**		
MPFBA	$^{13}C_4$ Perfluorobutanoic acid	$^{13}C_4HF_7O_2$
M5PFHxA	$^{13}C_5$ Perfluorohexanoic acid	$^{13}C_5{}^{12}C_1HF_{11}O_2$
M4PFHpA	$^{13}C_4$ Perfluoroheptanoic acid	$^{13}C_4{}^{12}C_3HF_{13}O_2$
M8PFOA	$^{13}C_8$ Perfluorooctanoic acid	$^{13}C_5C_8HF_{15}O_2$
M9PFNA	$^{13}C_9$ Perfluorononanoic acid	$^{13}C_5C_9HF_{17}O_2$

M6PFDA	$^{13}C_6$ Perfluorodecanoic acid	$^{13}C_6{}^{12}C_4HF_{19}O_2$
M7PFUnDA	$^{13}C_7$ Perfluoroundecanoic acid	$^{13}C_7{}^{12}C_4HF_{21}O_2$
MPFDoDA	$^{13}C_2$ Perfluorododecanoic acid	$^{13}C_2{}^{12}C_{10}HF_{23}O_2$
M3HFPO-DA	$^{13}C_3$ Hexafluoropropylene oxide dimer acid	$^{13}C_3{}^{12}C_3C_6HF_{11}O_3$
d3- N-MeFOSAA	$^{12}C_{11}{}^2H_3$ N-methyl perfluorooctanesulfonamidoacetic acid	$C_{11}{}^2H_3{}^1H_3$ $F_{17}NO_4S$
d5- N-EtFOSAA	$^{12}C_{12}{}^2H_5$ N-ethyl perfluorooctanesulfonamidoacetic acid	$C_{12}{}^2H_5{}^1H_3F_{17}NO_4S$
M3PFBS	$^{13}C_3$ Perfluorobutane sulfonate	$^{13}C_3{}^{12}C_1F_9SO_3H$
M3PFHxS	$^{13}C_3$ Perfluorohexane sulfonate	$^{13}C_3{}^{12}C_3F_{13}SO_3H$
M8PFOS	$^{13}C_8$ Perfluorooctane sulfonate	$^{13}C_8F_{17}SO_3H$
M2-4:2 FTS	$^{13}C_2$ 4:2 fluorotelomer sulfonic acid	$^{13}C_2{}^{12}C_4H_5F_9O_3S$
M2-6:2 FTS	$^{13}C_2$ 6:2 fluorotelomer sulfonic acid	$^{13}C_2{}^{12}C_6H_5F_{13}O_3S$
M2-8:2 FTS	$^{13}C_2$ 8:2 fluorotelomer sulfonic acid	$^{13}C_2{}^{12}C_8H_5F_{17}O_3S$

Table S4. UPLC-MS/MS instrument parameters for the quantification of target PFASs

UPLC-MS/MS conditions				
Instrument	Agilent 1290 infinity II UPLC coupled to an API 5500 triple-quadrupole mass spectrometer (AB SCIEX Inc., Framingham, MA, USA)			
Analytical column	Acquity BEH C18 column (100 mm × 2.1 mm, 1.7 μm, Waters, MA, USA)			
Column temperature	40 °C			
Injection volume	2 μL			
Mobile phase	2 mM ammonium acetate in water (A) and acetonitrile (B)			
Gradient	Time (min)	A (%)	B (%)	Flow rate (mL/min)
	0	70	30	0.3
	0.5	70	30	0.3
	10	0	100	0.3
	11	0	100	0.3
	11.2	70	30	0.3
	13	70	30	0.3

Other mass parameters	API 5500, AB Sciex<br>Ion Spray Voltage: -4.2 kV;<br>Curtain Gas: 20 psi;<br>Collision Gas: 9 psi;<br>Temperature: 300 °C;<br>Ion Source Gas 1: 50 psi<br>Ion Source Gas 2: 40 psi

Table S5. Optimal UPLC-MS/MS parameters for the target PFASs and internal standards

Analyte	Precursor ion	Product ion	DP[a]	CE[b]	Internal standard
PFBA	213	169[c]/147	-38	-11	$^{13}C_4$-PFBA
PFHxA	313	269[c]/119	-45	-11	$^{13}C_5$-PFHxA
PFHpA	363	319[c]/169	-40	-13	$^{13}C_4$-PFHpA
PFOA	413	369[c]/169	-60	-15	$^{13}C_8$-PFOA
PFNA	463	419[c]/219	-72	-14	$^{13}C_9$-PFNA
PFDA	513	469[c]/219	-57	-16	$^{13}C_6$-PFDA
PFUnDA	563	519[c]/269	-80	-18	$^{13}C_7$-PFUnDA
PFDoDA	613	569[c]/169	-90	-15	$^{13}C_2$-PFDoDA
PFBS	299	80[c]/99	-82	-67	$^{13}C_3$-PFBS
PFPeS	349	80[c]/99	-150	-70	$^{13}C_3$-PFHxS

PFHxS	399	80[c]/99	-100	-72	$^{13}C_3$-PFHxS
PFHpS	449	80[c]/99	-110	-99	$^{13}C_3$-PFHxS
PFOS	499	80[c]/99	-83	-101	$^{13}C_8$-PFOS
PFNS	549	80[c]/99	-117.2	-115	$^{13}C_8$-PFOS
N-MeFOSAA	570	419[c]/219	-80	-29	d_3-N-MeFOSAA
N-EtFOSAA	584	419[c]/219	-55	-28	d_3-N-EtFOSAA
6:2 Cl-PFESA	531	351[c]/83	-85	-36.3	$^{13}C_8$-PFOS
8:2 Cl- PFESA	631	451[c]/83	-139	-38	$^{13}C_8$-PFOS
8Cl-PFOS	515	99	-90	-93	$^{13}C_8$-PFOS
HFPO-DA	329	285	-37	-7	$^{13}C_3$-HFPO-DA
HFPO-TA	495	185[c]/119	-51	-20	$^{13}C_8$-PFOA
HFPO-TeA	661	185[c]/119	-58	-35	$^{13}C_8$-PFOA
4:2 FTS	327	307[c]/81	-98	-27	$^{13}C_2$-4:2 FTS

6:2 FTS	427	407[c]/81	-81	-33	$^{13}C_2$-6:2 FTS
8:2 FTS	527	507[c]/81	-60	-35	$^{13}C_2$-8:2 FTS
$^{13}C_4$-PFBA	217	172	-40	-12	
$^{13}C_5$-PFHxA	318	273	-51	-14	
$^{13}C_4$-PFHpA	367	322	-61	-13	
$^{13}C_8$-PFOA	421	376	-45	-14	
$^{13}C_9$-PFNA	472	427	-62	-16	
$^{13}C_6$-PFDA	519	474	-75	-17	
$^{13}C_7$-PFUnDA	570	525	-40	-17	
$^{13}C_2$-PFDoDA	615	570	-65	-18	
$^{13}C_3$-HFPO-DA	332	287	-35	-9	
d_3-N-MeFOSAA	573	419	-86	-28	
d_3-N-EtFOSAA	589	419	-107	-29	

$^{13}C_3$-PFBS	302	80	-52	-59
$^{13}C_3$-PFHxS	402	80	-168	-85
$^{13}C_8$-PFOS	507	80	-180	-106
$^{13}C_2$-4:2 FTS	329	309[c]/81	-85	-29
$^{13}C_2$-6:2 FTS	429	409[c]/81	-85	-34
$^{13}C_2$-8:2 FTS	529	509[c]/81	-83	-38

[a] Decluster potential.

[b] Collision energy.

[c] Quantitative ion.

Table S6. The matrix spike recoveries (MSRs ± SD) of individual PFASs in crops and soil.

Compounds	Soil	Crops
**PFCAs**		
PFBA	88.2 ± 9.4%	100.7 ± 6%
PFHxA	101 ± 9.1%	95.5 ± 2.1%
PFHpA	89.3 ± 3.8%	99.5 ± 0.8%
PFOA	85.5 ± 1.0%	100.7 ± 1.7%
PFNA	85.2 ± 2.8%	97.4 ± 2.9%
PFDA	91.8 ± 2.2%	93.6 ± 6.5%
PFUnDA	89.0 ± 1.9%	98.8 ± 3.9%
PFDoDA	90.9 ± 5.4%	96.0 ± 3.0%
**PFSAs**		
PFBS	94.2 ± 5.9%	94.1 ± 14.0%
PFPeS	90.0 ± 7.0%	100.9 ± 2.7%
PFHxS	107.5 ± 9.8%	93.1 ± 5.8%
PFHpS	95.5 ± 8.2%	95.0 ± 6.5%
PFOS	84.4 ± 4.8%	96.7 ± 0.9%
PFNS	83.6 ± 3.4%	91.1 ± 3.9%
**Precursor substances**		
N-MeFOSAA	101.6 ± 3.7%	99.1 ± 7.2%

N-EtFOSAA	87.2 ± 6.7%	93.2 ± 2.0%
**Alternatives**		
6:2 Cl-PFESA	82.9 ± 3.4%	95.0 ± 3.8%
8:2 Cl- PFESA	79.9 ± 8.6%	104.5 ± 2.7%
8Cl-PFOS	80.0 ± 8.0%	86.2 ± 3.9%
HFPO-DA	91.0 ± 2.8%	93.7 ± 8.5%
HFPO-TA	108.3 ± 1.3%	105 ± 3.5%
HFPO-TeA	95.4 ± 10.7%	103.2 ± 0.4%
4:2 FTS	91.7 ± 3.6%	106.6 ± 4.7%
6:2 FTS	80.5 ± 3.1%	102.7 ± 1.4%
8:2 FTS	79.9 ± 8.6%	104.5 ± 2.7%

Table S7. Reference daily crop intake (g/d), body weight (BW, kg) and Maximum moisture content of different crops for three age groups in urban and rural areas of China.

	Toddlers (2-5 y)		Children and teenagers (6-17 y)		Adults (>18 y)		Moisture content
	Urban	Rural	Urban	Rural	Urban	Rural	
Leafy vegetables	143.9	120.9	194.8	175.2	276.2	285.6	95.8%
Solanaceae	143.9	120.9	194.8	175.2	276.2	285.6	95.6%
Gramineae	143.9	120.9	194.8	175.2	276.2	285.6	71.3%
Cucurbitaceae	143.9	120.9	194.8	175.2	276.2	285.6	96.6%
Leguminosae	143.9	120.9	194.8	175.2	276.2	285.6	91.9%
BW	18	17	41.5	36.1	63.7	61.9	

HRs	HR (Leafy vegetables)						HR (Solanaceae)						HR (Gramineae)					
Age	2-5 y		6-17 y		>18 y		2-5 y		6-17 y		>18 y		2-5 y		6-17 y		>18 y	
Area	U	R	U	R	U	R	U	R	U	R	U	R	U	R	U	R	U	R
PFBA	0.009	0.008	0.006	0.006	0.005	0.005	0.012	0.011	0.007	0.007	0.007	0.007	0.004	0.003	0.003	0.002	0.002	0.002
PFHxA	0.003	0.002	0.002	0.002	0.002	0.002	ND	ND	ND	ND	ND	ND	ND	ND	ND	ND	ND	ND
PFOA	0.035	0.031	0.020	0.021	0.019	0.020	ND	ND	ND	ND	ND	ND	ND	ND	ND	ND	ND	ND
PFBS	ND	ND	ND	ND	ND	ND	0.011	0.010	0.007	0.007	0.006	0.006	ND	ND	ND	ND	ND	ND
HFPO-DA	0.004	0.003	0.002	0.002	0.002	0.002	0.001	0.001	0.001	0.001	0.001	0.001	ND	ND	ND	ND	ND	ND
HI	0.051	0.045	0.030	0.031	0.027	0.030	0.025	0.022	0.014	0.015	0.013	0.014	0.004	0.003	0.003	0.002	0.002	0.002

Table S8. HRs from consumption of contaminated crops by urban and rural residents of different age groups

Continue:

HRs	HR (Cucurbitaceae)						HR (Leguminosae)					
Age	2-5 y		6-17 y		>18 y		2-5 y		6-17 y		>18 y	
Area	U	R	U	R	U	R	U	R	U	R	U	R
PFBA	0.001	0.001	0.001	0.001	0.001	0.001	0.017	0.015	0.010	0.010	0.009	0.010
PFHxA	ND	ND	ND	ND	ND	ND	ND	ND	ND	ND	ND	ND
PFOA	0.002	0.002	0.001	0.001	0.001	0.001	ND	ND	ND	ND	ND	ND
PFBS	ND	ND	ND	ND	ND	ND	ND	ND	ND	ND	ND	ND
HFPO-DA	0.001	0.001	ND	ND	ND	ND	0.001	0.001	0.001	0.001	0.001	0.001

HI	0.004	0.003	0.002	0.002	0.002	0.002	0.019	0.017	0.011	0.012	0.010	0.011

Note: ND, nondetectable.

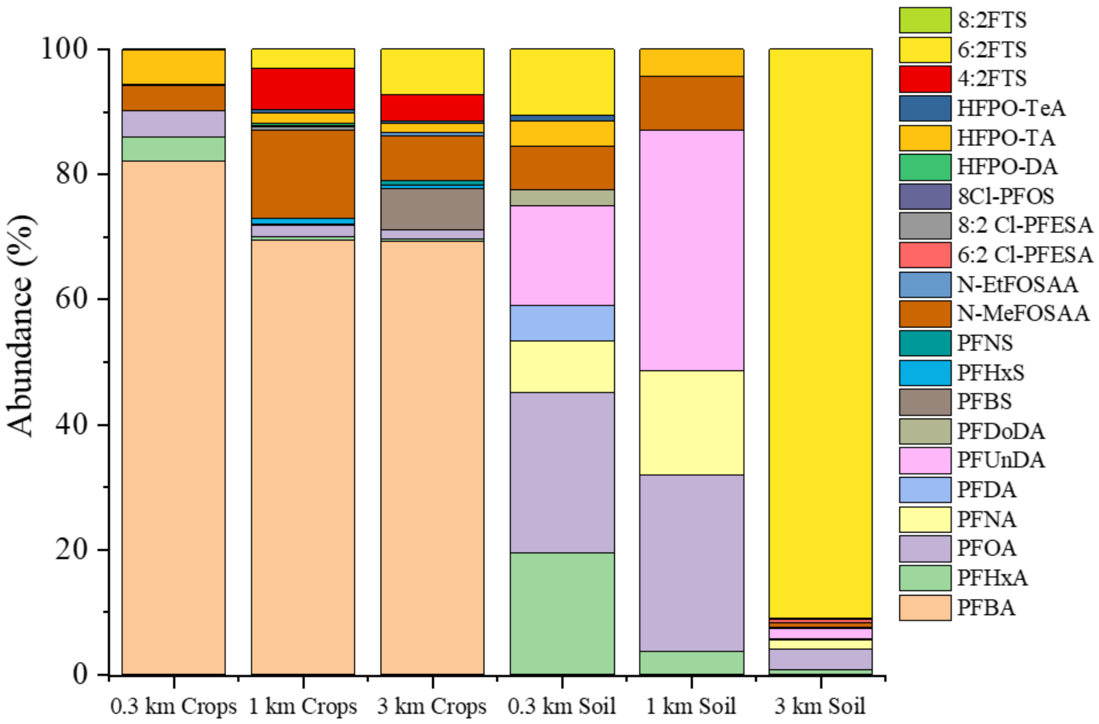

Figure S1. Proportion of different PFASs in soil and crop samples.
